# Expression of microRNAs in patients with gestational diabetes mellitus: a systematic review and meta-analysis

**DOI:** 10.1007/s00592-022-02005-8

**Published:** 2022-12-17

**Authors:** Jianhua Li, Bei Gan, Lin Lu, Lihong Chen, Jianying Yan

**Affiliations:** 1 Department of Obstetrics and Gynecology, The First Affiliated Hospital of Fujian Medical University, Fuzhou, 350005 Fujian China; 2 Department of Obstetrics, Fujian Maternity and Child Health Hospital, College of Clinical Medicine for Obstetrics & Gynecology and Pediatrics, Fujian Medical University, Fuzhou, China

**Keywords:** Expression, miRNA, Gestational diabetes mellitus, Meta-analysis

## Abstract

**Background:**

MicroRNAs (miRNA) are noncoding RNAs that play a central role in governing various physiological and pathological processes. There are few studies on miRNA involvement in gestational diabetes mellitus (GDM). In this study, we performed a meta-analysis of the miRNA expression profiling from GDM patients.

**Methods:**

Guided by the Preferred Reporting Items for Systematic Review and Meta-Analysis Protocols, we performed a systematic search of the PubMed, Cochrane Library, and EMBASE databases from inception to December 20, 2021, to retrieve the original research studies. All the relevant data were retrieved, analyzed, and summarized.

**Results:**

Six studies (252 GDM cases and 309 controls) were included and analyzed. The six studies reported the expressions of 21 miRNAs in GDM cases. Of the 21 miRNAs, 12 miRNAs were found to be upregulated, and two were downregulated. The top three most consistently reported upregulated miRNAs were miR-16-5p (mean differences of fold change are 1.25, 95% CI = 0.04–2.46, *P* = 0.040), miR-19a-3p (mean differences of fold change are 2.90, 95% CI = 1.45–4.35, *P* = 0.001), and miR-19b-3p (mean differences of fold change are 3.10, 95% CI = 0.94–5.25, *P* = 0.005). miR-155-5p and miR-21-3p were found to be downregulated.

**Conclusions:**

The results indicate that several miRNAs may be used as markers for diabetes gestational diabetes mellitus. In the future, more studies are needed to validate the findings of our study.

**Supplementary Information:**

The online version contains supplementary material available at 10.1007/s00592-022-02005-8.

## Introduction

In the past decades, without effective prevention strategies, the incidence of gestational diabetes mellitus (GDM) is rapidly increasing, resulting in clinical and public health concerns [1, 2]. GDM is characterized by the potential for severe GDM-related pregnancy complications and might to negative economic impact [3]. The etiology of GDM is complex and is decided by genetic and environmental factors implicated in mechanistic and epidemiological studies [4].

MicroRNAs (miRNAs), a new class of noncoding RNAs of between 20 and 25 nucleotides in length, play important roles in posttranscriptional gene regulation and multiple cellular processes [5]. Gene expression profiling studies have demonstrated alterations in miRNA expression in a wide range of human diseases. Previous studies have linked miRNA dysregulation as a causal factor in disease progression, including many cancer types, cardiovascular diseases, and metabolic diseases [6–8]. Several studies have suggested that there is a selective expression of miRNAs that may be associated with diabetic conditions [9, 10]. In recent years, miRNAs have emerged as promising diagnostic and therapeutic tools due to their association with GDM [8, 11]. For example, miR‐137 displays high expressions, whereas its target gene (fibronectin type III domain containing 5) is downregulated among women with GDM [12]. A previous study also reported that a low level of miR‐21‐3p in the blood leucocytes of women might increase the risk of GDM [13]. However, dozens of miRNAs are identified to be differentially expressed; the miRNAs can be either over- or under-expressed. Given the large number of candidate signatures, a study to summarize the expression of miRNAs in GDM is needed. In this study, we aimed to investigate the expression of miRNAs in GDM systemically.

## Materials and methods

### Information sources and literature search strategies

A systematic search for the expression profiles of miRNAs was performed by using PubMed, Cochrane Library, and EMBASE database up to December 20, 2021. The individual and combined keywords of “MicroRNAs,” “miRNAs,” “gestational diabetes mellitus,” and “GDM” were used. Detailed search strategies in the three databases are presented in the **Supplementary Materials**. References of the included articles were also screened to check all the available articles. For the search of the gray literature, Google Scholar was used to identify any articles not included in the databases above.

This review was conducted according to the guidelines of the Preferred Reporting Items for Systematic Reviews and Meta-analyses (PRISMA) statement [14].

### Eligible criteria

Articles were included if they met the following criteria:

1. Studies evaluated the expression of blood (i.e., serum or plasma) miRNAs between healthy individuals and GDM in human and animal studies;
2. Investigations used microarray and/or real-time polymerase chain reaction (RT-PCR) to evaluate the expression of miRNAs and carried out fold changes in gene expression of at least one miRNA;
3. necessary data extracted from original studies;
4. articles are written only in English;
5. only the study providing more detailed information was included if the population was reported in duplicate.

Articles under the following criteria were excluded:

1. if no fold changes were reported;
2. if no disease/experimental group was considered;
3. studies reporting on the miRNA expression from in vitro cell lines or animals;
4. reviews, case reports, abstracts or posters for conferences, personal opinions, and book chapters;
5. studies published in languages other than English.

### Data extraction and analyses

Two authors (JHL and BG) independently reviewed all full-text articles, and they initially resolved any disagreements through discussions. If the two authors did not agree, a third author (LHC) made the final decision. The needed information was extracted using a customized and standardized form.

For each included study, the following information was extracted: the author and year of publication, country, sample size, definition of GDM and control, ages of GDM and control, BMI of GDM and control, and the fold changes.

The primary outcome considered was the fold changes of miRNA expression in GDM.

### Risk of bias and quality assessment of selected studies

The assessment of the study quality is evaluated by the Newcastle–Ottawa Scale (NOS) [15], a risk of bias assessment tool for observational studies that the Cochrane Collaboration recommends. Two authors (CGP and FA) independently evaluated the included studies, and disagreements were resolved by discussion to produce final scores. The NOS assigns up to a maximum of nine points for the least risk of bias in three domains: (1) selection of study groups (four points); (2) comparability of groups (two points); and (3) ascertainment of exposure and outcomes (three points) for case–control and cohort studies, respectively.

### Statistical analysis

The fold changes with corresponding 95% confidence intervals (CIs) in GDM and control groups were used to quantify the miRNA expression in GDM. The fold changes in each included study were combined using a random effect model, and the statistically significant difference was determined using *P* values from the pooled fold changes. Study heterogeneity was calculated with I^2^ and Cochran’s Q statistics. The Begg rank correlation [16] and Egger weighted regression methods [17] were used to assess the publication bias (*P* < 0.05 was considered indicative of a statistically significant publication bias). Review Manager (version 5.3, The Cochrane Collaboration, Oxford, UK) was used to generate forest plots and statistical analyses. The Begg and Egger tests were assessed by STATA 15.0 (Stata Corporation, College Station, TX, USA). A two-sided *P* value of < 0.05 was considered significant for all analyses.

## Results

### Search results and characteristics of studies included in the meta-analysis

**Fig. 1:**
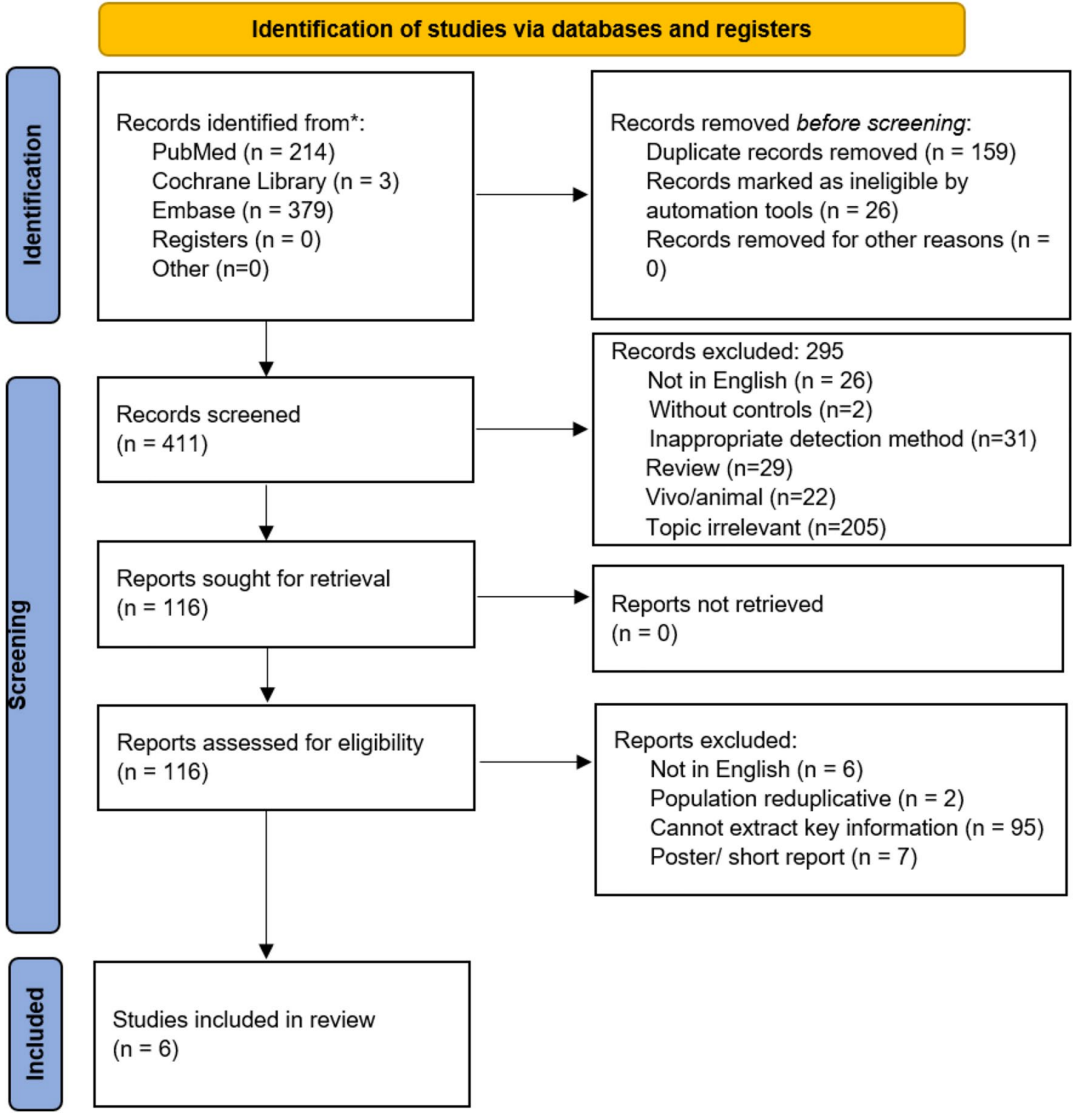
Flowchart of the study selection

Initial screening of electronic databases yielded a total of 596 articles; 159 were excluded due to the elimination of duplicated studies, and then 411 titles or abstracts were further evaluated. The screening of titles and abstracts resulted in 116 potentially relevant articles being selected. After retrieving the 116 full-length manuscripts, ultimately, six studies fulfilled the inclusion and were assessed in this systematic review and meta-analysis [13, 18–22]. The flowchart of the studies enrolled in the current study can be found in Fig. 1.

### Characteristics of included studies

The characteristics of the included studies are shown in Table 1. The six included studies were published between 2018 and 2020. A total of 252 GDM cases and 309 controls were included. The studies were conducted one in South Africa, two in Turkey, one in Canada, and two in China,. The controls were healthy pregnant women; most of them were matched by gestational weeks with corresponding GDM cases. Gestational age (GA) was determined from the last menstrual period and verified during the routine first-trimester ultrasound measurement of the fetal crown–rump length.

**Table 1:** Characteristics of the included studies

Study	Country	Sample size		Definition of control	Matching factors	Age (years)		BMI (kg/m^2^)		Gestational age (weeks)		Sources	Definition OF GDM	Platform
		GDM	Control			GDM	Control	GDM	Control	GDM	Control			
[21]	South African	28	53	Without GDM	Age and BMI	29.5 ± 6.2	28.6 ± 6.4	28.1 (23.9–31.3)	26.2 (21.9–29.8)	26.0 (24.0–28.0)	27.0 (25.0–28.0)	Serum	IADPSG	Quantitative real-time PCR
[19]	Turkey	19	28	Healthy pregnant women	Gestational weeks	30.4 ± 4.6	28.1 ± 5.8	30.7 ± 4.1	27.1 ± 2.8	33.5 ± 3.6	33 ± 4.1	Plasma	IADPSG	Real-time quantitative PCR
[19]	Canada	23	46	Healthy pregnant women	Gestational weeks	29.8 ± 5.3	27.9 ± 4.4	28.2 ± 7.2	24.5 ± 4.7	10.5 ± 2.5	10.6 ± 2.4	Serum	SOGC	Quantitative real-time PCR
[20]	Turkey	14	27	Healthy pregnant women	Gestational weeks	30.4 ± 4.4	27.9 ± 5.5	30.6 ± 4.0	27.1 ± 2.9	33.5 ± 3.5	33.1 ± 4.1	Plasma	IADPSG	Real-time quantitative PCR
[18]	China	68	55	Healthy pregnant women	Gestational weeks	32.65 ± 4.63	31.27 ± 4.01	23.06 ± 3.56	21.88 ± 2.93	39.09 ± 1.11	38.98 ± 1.05	Serum	GDM by the American Diabetes Association on in 2012	Real-time quantitative PCR
[22]	China	100	100	Healthy pregnant women	NA	NA	NA	28.41 ± 2.18	23.56 ± 1.52	NA	NA	Serum	Oral glucose tolerance test at 24–28 weeks of gestation	Real-time quantitative PCR

*GDM* gestational diabetes mellitus, *BMI* body mass index, *IADPSG* International Association of Diabetes and Pregnancy, *SOGC* the guidelines of the Society of Obstetricians and Gynaecologists of Canada, *PCR* Polymerase Chain Reaction, *NA* not available

GDM cases and controls have similar ages, which were all about 30 years old. GDM cases had slightly higher body mass index than controls. All miRNA expression was detected in blood samples (either serum or plasma) using quantitative real-time PCR.

Three of the studies defined GDM following the International Association of Diabetes and Pregnancy. GDM was made when 1 of the following plasma glucose values in the oral glucose tolerance test was met or exceeded: fasting plasma glucose 92 mg/dL (5.1 mmol/L), 1-h plasma glucose 180 mg/dL (10.0 mmol/L), or 2-h plasma glucose 153 mg/dL (8.5 mmol/L) [23]. One study defended GDM according to the guidelines of the Society of Obstetricians and Gynaecologists of Canada: fasting venous plasma glucose level > 5.3 mmol/L, 1-h plasma glucose level > 10.6 mmol/L, or 2-h plasma glucose level > 9.0 mmol/L.

### Quality assessment of studies

Newcastle–Ottawa Scales for the eligible studies are presented in **Supplementary Table 1**. All included studies are found to exhibit an acceptable quality. Three studies, two, and one were evaluated as eight points, seven points, and six points, respectively.

### Fold changes of miRNA expression in GDM

The six studies reported the fold changes of 21 miRNAs in GDM cases, including miR-16-5p, miR-17-5p, miR-19a-3p, miR-19b-3p, miR-20a-5p, miR-29a-3p, miR-132-3p, miR-222-3p, miR-21-3p, miR-155-5p, miR-29b-3p, miR-122-5p, miR-1323, miR-182-3p, miR-210-3p, miR-520h, miR-136-5p, miR-342-3p, miR-494-3p, and miR-517-5p with the means of the fold changes ranged from 0.67 (miR-21-3p) to 25.92 (miR-29a-3p) in GDM and from 0.96 (miR-517-5p) to 20.13 (miR-29a-3p) in controls. The detailed results on fold changes of 20 miRNAs are presented in Table 2.

**Table 2:** MicroRNAs fold changes in gestational diabetes mellitus and controls

Study	MicroRNA	GDM	Control
[21]	miR-16-5p	4.2 (3.1)	3.3 (3.2)
	miR-17-5p	10.7 (3.4)	9.4 (3.5)
	miR-19a-3p	9.4 (3.3)	8.0 (3.2)
	miR-19b-3p	7.7 (3.6)	6.8 (3.1)
	miR-20a-5p	9.1 (3.1)	7.7 (3.5)
	miR-29a-3p	6.6 (4.1)	5.6 (3.4)
	miR-132-3p	11.5 (3.0)	10.2 (3.0)
	miR-222-3p	9.3 (2.6)	7.9 (2.9)
	miR-21-3p	0.67 (0.54)	1.31 (1.13)
	miR-155-5p	0.98 (0.79)	1.29 (0.97)
[13]	miR-16-5p	3.14 (5.43)	1.29 (0.97)
[19]	miR-29a-3p	1.43 (0.22)	0.97 (0.09)
	miR-29b-3p	1.40 (0.22)	1.42 (0.17)
	miR-122-5p	0.98 (0.09)	0.95 (0.10)
	miR-132-3p	1.30 (0.12)	0.94 (0.08)
	miR-1323	1.54 (0.26)	0.97 (0.13)
	miR-182-3p	1.51 (0.20)	0.99 (0.11)
	miR-210-3p	1.67 (0.39)	0.97 (0.08)
	miR-520 h	1.2 (0.2)	0.80 (0.07)
	miR-136-5p	1.48 (0.24)	0.99 (0.10)
	miR-342-3p	1.50 (0.18)	0.95 (0.11)
	miR-494-3p	1.40 (0.23)	1.00 (0.12)
	miR-517-5p	1.30 (0.22)	0.96 (0.10)
[20]	miR-16-5p	5.60 (9.97)	2.35 (2.05)
	miR-155-5p	1.09 (0.91)	1.63 (1.46)
[18]	miR-29a-3p	25.92 (5.01)	20.13 (3.4)
	miR-29b-3p	22.49(3.46)	17.28 (3.24)
[22]	miR-19a-3p	4.0 (0.92) ^a^	NA
	miR-19b-3p	4.77 (1.55) ^a^	NA

*GDM* gestational diabetes mellitus, *NA* not available

^a^Mean difference

**Fig. 2:**
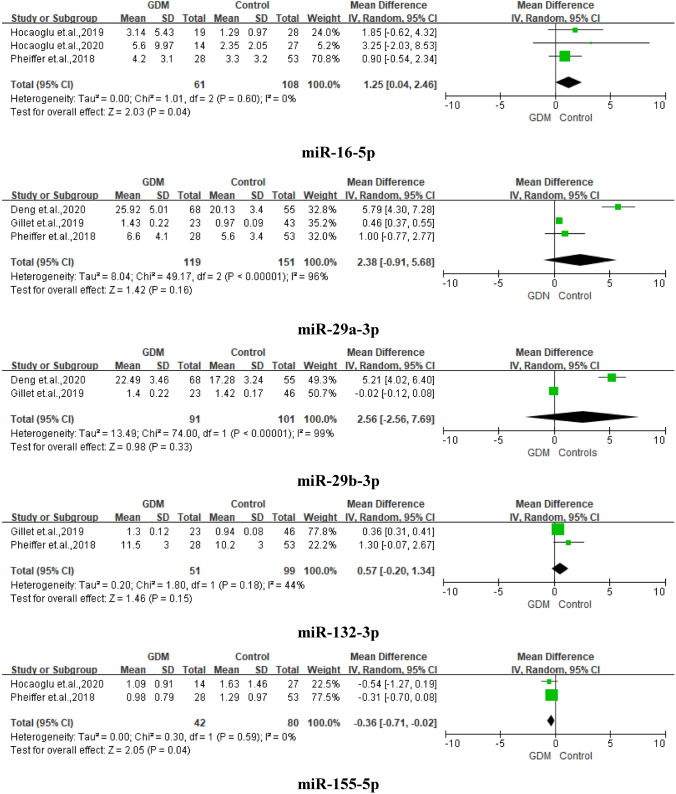
Forest plots for summarized fold changes in gestational diabetes mellitus and controls

Table 3 shows the miRNAs with significant expression differences in gestational diabetes mellitus. Of the 20 miRNAs, miR-16-5p, miR-19a-3p, miR-19b-3p, miR-155-5p, miR-29a-3p, miR-29b-3p, and miR-132-3p were reported more than once and were pooled. The forest plots are shown in Fig. 2.

**Table 3:** MicroRNAs with significant expression differences in gestational diabetes mellitus

MicroRNA	Number of included studies	Mean differences^a^	*P* value	Heterogeneity (*I*^2^)	Publication bias	
					Bagger	Egger
*Upregulated*						
miR-16-5p	3	1.25 (0.04–2.46)	0.040	0%	0.41	0.34
miR-19a-3p	2	2.90 (1.45–4.35)	0.001	51%	0.61	0.17
miR-19b-3p	2	3.10 (0.94–5.25)	0.005	57%	0.19	0.17
miR-20a-5p	1	9.1 (3.1)/7.7 (3.5)	0.038	NA	NA	NA
miR-222-3p	1	9.3 (2.6)/7.9 (2.9)	0.027	NA	NA	NA
miR-122-5p	1	0.98 (0.09)/0.95 (0.10)	0.010	NA	NA	NA
miR-1323	1	1.54 (0.26)/0.97 (0.13)	0.030	NA	NA	NA
miR-182-3p	1	1.51 (0.20)/0.99 (0.11)	0.010	NA	NA	NA
miR-210-3p	1	1.67 (0.39)/0.97 (0.08)	0.020	NA	NA	NA
miR-520 h	1	1.2 (0.2)/0.80 (0.07)	0.030	NA	NA	NA
miR-136-5p	1	1.48 (0.24)/0.99 (0.10)	0.030	NA	NA	NA
miR-342-3p	1	1.50 (0.18)/0.95 (0.11)	0.008	NA	NA	NA
*Downregulated*						
miR-155-5p	2	− 0.36 (− 0.71 to − 0.02)	0.040	0%	0.14	0.28
miR-21-3p	1	0.67 (0.54)/1.31 (1.13)	0.001	NA	NA	NA

*NA* not available

^a^Mean differences for data on MicroRNA more than one study

Twelve miRNAs were found to be upregulated with mean differences ranged from 1.25 (miR-16-5p, 95% CI = 0.04–2.46, *P* = 0.040) to 3.10 (miR-19b-3p, 95% CI = 0.94–5.25, *P* = 0.005). Two were downregulated, miR-155-5p with mean differences as -0.36 (95% CI = − 0.71 to − 0.02, *P* = 0.040) and miR-21-3p (*P* = 0.001).

The seven miRNAs without expression differences in gestational diabetes mellitus are presented in Table 4.

**Table 4:** MicroRNAs without expression differences in gestational diabetes mellitus

MicroRNA	Number of included studies	Mean differences	*P* value	Heterogeneity (*I*^2^)	Publication bias	
					Bagger	Egger
miR-29a-3p	3	2.38 (− 0.91 to 5.68)	0.160	96%	0.69	0.51
miR-29b-3p	3	2.56 (− 2.56 to 7.69)	0.330	99%	0.94	0.81
miR-132-3p	2	0.57 (− 0.20 to 1.34)	0.150	44%	0.64	0.51
miR-17-5p	1	10.7 (3.4)/9.4 (3.5)	0.121	NA	NA	NA
miR-132-3p	1	1.30 (0.12)/0.94 (0.08)	0.120	NA	NA	NA
miR-494-3p	1	1.40 (0.23)/1.00 (0.12)	0.100	NA	NA	NA
miR-517-5p	1	1.30 (0.22)/0.96 (0.10)	0.120	NA	NA	NA

*NA* not available

### Publication bias

The analysis did not find potential publication bias among the included trials according to Begg rank correlation analysis and Egger weighted regression analysis (*P* > 0.05). The detailed potential publication bias can be found in Tables 3, 4**.**

## Discussion

To the best of our knowledge, the current meta-analysis is the first systematic review and meta-analysis study summarizing the expression of miRNAs in GDM. Six studies (252 GDM cases and 309 controls) were included and analyzed. The six studies reported the fold changes of 21 miRNAs in GDM cases. Of the 21 miRNAs, 12 miRNAs were found to be upregulated, and two were downregulated. The top three most consistently reported upregulated miRNAs were miR-16-5p, miR-19a-3p, and miR-19b-3p.

Several miRNAs have attracted interest in recent years as biomarkers of metabolic disease and cancers [24, 25]. In our study, miR-16-5p, miR-19a-3p, and miR-19b-3p were upregulated in GDM. A study by Hocaoglu et al. [20] reported that increased miR-16-5p expression is associated with PCOS in pregnancy. miR-16-5p was also reported to be one of the most abundant miRNAs in several types of cancer [26–28]. A recent study also found miR-16-5p implicated in type 1 diabetes mellitus [29]. Consequently, the miR-16-5p has the potential to identify suitable miRNAs for GDM and even type 1 diabetes mellitus and cancer diagnosis. Similarly, the overexpression of miR-19a-3p promoted cell proliferation and insulin secretion [30]. A direct target gene of miR‑19a‑3p, the suppressor of cytokine signaling 3 (SOCS3), was inversely correlated with the miR‑19a‑3p level. The SOCS3 contributes to the dysfunction of pancreatic β cells, suggesting that miR‑19a‑3p plays an important role in β cell function. The miR-19a-3p/SOCS3 axis may there be a potential therapeutic target for diabetes [30, 31]. Moreover, several of the remaining miRNAs were also reported to have the potential to be GDM or diabetes diagnostic and prognostic markers, i.e., miR-21-3p, miR-210-3p, miR-122-5p, etc. [19, 32–36]

This study highlights the meaningful identification of miRNA-based diagnostic and prognostic markers for the most prevalent GDM. Our study also indicates a new insight into the putative functions of miRNAs and may provide evidence to delineate the mechanisms through which they are released into the bloodstream. We suggested several promising miRNAs that have an average of more than twofold change. Their potential targets may provide a clue to the role of miRNAs in understanding the underlying mechanisms of GDM.

There are several factors needed to pay close attention to when identifying miRNAs as GDM candidate clinical biomarkers. Firstly, the biological mechanism should be well understood. A single miRNA may have many targets, and also, a specific mRNA may be regulated by multiple different miRNAs. Second, there should be sufficient information about their pattern of expression in different kinds of specimens in target populations. Third, rigorous validation and demonstration of reproducibility in an independent population are necessary to confirm the predictive value of miRNAs.

The strength of our study is the first systematic assessment of miRNAs expression in GDM. However, although with a large of included studies, it is necessary to consider the limitations of the present meta-analysis while interpreting the results. First, the included studies assessed various miRNAs to observe the expression of miRNAs in GDM. The majority of them cannot be pooled using a meta-method because they were reported once. Our study, therefore, highlighted the need for future studies on the topic. At the same time, our study also highlighted the importance of study design regarding the comparability among studies conducted by various researchers. Second, the number of pooled results was limited. The current results might be affected by environmental factors, which can only partially annotate the expressions of miRNAs, and the representativeness might be weakened. Third, due to the insufficient information in each study, we could not pool subgroup analysis. Fourth, potential language bias might exist because our literature search included only articles published in English. Fifthly, publication bias cannot be assessed for all analyses as a limited number of outcomes were reported once.

In conclusion, our meta-analysis provided pooled results based on six studies and summarized a data set of 252 GDM cases and 309 controls. The current study highlighted several miRNAs which have the potential to be diagnostic and prognostic markers. In the future, efforts must be made to focus on the topic.

## Supplementary Information

Below is the link to the electronic supplementary material.

Supplementary file1 (DOCX 18 KB)

## Data Availability

All data generated or analyzed during this study are included in this published article.

## References

[1] Alfadhli EM (2015). Gestational diabetes mellitus. Saudi Med J.

[2] Chiefari E, Arcidiacono B, Foti D, Brunetti A (2017). Gestational diabetes mellitus: an updated overview. J Endocrinol Invest.

[3] Johns EC, Denison FC, Norman JE, Reynolds RM (2018). Gestational diabetes mellitus: mechanisms, treatment, and complications. Trends Endocrinol Metab.

[4] Juan J, Yang H (2020). Prevalence, prevention, and lifestyle intervention of gestational diabetes mellitus in China. Int J Environ Res Public Health.

[5] Bushati N, Cohen SM (2007). microRNA functions. Annu Rev Cell Dev Biol.

[6] Hammond SM (2015). An overview of microRNAs. Adv Drug Deliv Rev.

[7] Wojciechowska A, Braniewska A, Kozar-Kaminska K (2017). MicroRNA in cardiovascular biology and disease. Adv Clin Exp Med.

[8] Vasu S, Kumano K, Darden CM, Rahman I, Lawrence MC, Naziruddin B (2019). MicroRNA signatures as future biomarkers for diagnosis of diabetes states. Cells.

[9] Wu B, Miller D (2017). Involvement of MicroRNAs in diabetes and Its complications. Methods Mol Biol.

[10] Zhang Y, Bai R, Liu C (2019). MicroRNA single-nucleotide polymorphisms and diabetes mellitus: a comprehensive review. Clin Genet.

[11] Dias S, Pheiffer C, Abrahams Y, Rheeder P, Adam S (2018). Molecular biomarkers for gestational diabetes mellitus. Int J Mol Sci.

[12] Peng HY, Li MQ, Li HP (2019). MiR-137 restricts the viability and migration of HTR-8/SVneo cells by downregulating FNDC5 in gestational diabetes mellitus. Curr Mol Med.

[13] Hocaoglu M, Demirer S, Senturk H, Turgut A, Komurcu-Bayrak E (2019). Differential expression of candidate circulating microRNAs in maternal blood leukocytes of the patients with preeclampsia and gestational diabetes mellitus. Pregnancy Hypertens.

[14] Page MJ, McKenzie JE, Bossuyt PM (2021). The PRISMA 2020 statement: an updated guideline for reporting systematic reviews. BMJ.

[15] G W The Newcastle Ottawa Scale (NOS) for assessing the quality of non-randomised studies in meta-analysis. In: Proceedings of the Third Symposium on Systematic Reviews, England, 2000. Oxford,

[16] Begg CB, Mazumdar M (1994). Operating characteristics of a rank correlation test for publication bias. Biometrics.

[17] Egger M, Davey Smith G, Schneider M, Minder C (1997). Bias in meta-analysis detected by a simple, graphical test. BMJ.

[18] Deng L, Huang Y, Li L, Chen H, Su J (2020). Serum miR-29a/b expression in gestational diabetes mellitus and its influence on prognosis evaluation. J Int Med Res.

[19] Gillet V, Ouellet A, Stepanov Y (2019). miRNA Profiles in extracellular vesicles from serum early in pregnancies complicated by gestational diabetes mellitus. J Clin Endocrinol Metab.

[20] Hocaoglu M, Demirer S, Loclar Karaalp I (2021). Identification of miR-16-5p and miR-155-5p microRNAs differentially expressed in circulating leukocytes of pregnant women with polycystic ovary syndrome and gestational diabetes. Gynecol Endocrinol.

[21] Pheiffer C, Dias S, Rheeder P, Adam S (2018). Decreased expression of circulating miR-20a-5p in South African women with gestational diabetes mellitus. Mol Diagn Ther.

[22] Wang F, Zhang X, Zhou H (2019). Role of cell free microRNA-19a and microRNA-19b in gestational diabetes mellitus patients. Biotech.

[23] Metzger BE, Gabbe SG, Persson B (2010). International association of diabetes and pregnancy study groups recommendations on the diagnosis and classification of hyperglycemia in pregnancy. Diabetes Care.

[24] Condrat CE, Thompson DC, Barbu MG (2020). miRNAs as biomarkers in disease: latest findings regarding their role in diagnosis and prognosis. Cells.

[25] To KK, Tong CW, Wu M, Cho WC (2018). MicroRNAs in the prognosis and therapy of colorectal cancer: from bench to bedside. World J Gastroenterol.

[26] Munson PB, Hall EM, Farina NH, Pass HI, Shukla A (2019). Exosomal miR-16-5p as a target for malignant mesothelioma. Sci Rep.

[27] Chava S, Reynolds CP, Pathania AS (2020). miR-15a-5p, miR-15b-5p, and miR-16-5p inhibit tumor progression by directly targeting MYCN in neuroblastoma. Mol Oncol.

[28] Ruan L, Qian X (2019). Biosci Rep.

[29] Gao X, Zhao S (2020). miRNA-16-5p inhibits the apoptosis of high glucose-induced pancreatic beta cells via targeting of CXCL10: potential biomarkers in type 1 diabetes mellitus. Endokrynol Pol.

[30] Li Y, Luo T, Wang L, Wu J, Guo S (2016). MicroRNA-19a-3p enhances the proliferation and insulin secretion, while it inhibits the apoptosis of pancreatic beta cells via the inhibition of SOCS3. Int J Mol Med.

[31] Chen Y, Wang W, Chen Y, Tang Q, Zhu W, Li D, Liao L (2019). MicroRNA-19a-3p promotes rheumatoid arthritis fibroblast-like synoviocytes via targeting SOCS3. J Cell Biochem.

[32] Bai X, Luo Q, Tan K, Guo L (2020). Diagnostic value of VDBP and miR-155-5p in diabetic nephropathy and the correlation with urinary microalbumin. Exp Ther Med.

[33] Cheng B, Li JY, Li XC, Wang XF, Wang ZJ, Liu J, Deng AP (2018). MiR-323b-5p acts as a novel diagnostic biomarker for critical limb ischemia in type 2 diabetic patients. Sci Rep.

[34] Jin ZQ (2021). MicroRNA targets and biomarker validation for diabetes-associated cardiac fibrosis. Pharmacol Res.

[35] Li X, Guo C, Chen Y, Yu F (2021). Long noncoding RNA SNHG16 regulates E2F1 expression by sponging miR-20a-5p and aggravating proliferative diabetic retinopathy. Can J Physiol Pharmacol.

[36] Shao M, Yu M, Zhao J, Mei J, Pan Y, Zhang J, Wu H, Yu M, Liu F, Chen G (2020). miR-21-3p regulates AGE/RAGE signalling and improves diabetic atherosclerosis. Cell Biochem Funct.

