# Supplementary material for: Expression of microRNAs in patients with gestational diabetes mellitus: a systematic review and meta-analysis
Source: Acta Diabetol. 2022 Dec 17;60(4):461–9. doi: 10.1007/s00592-022-02005-8 (PMC10033571; doi:10.1007/s00592-022-02005-8)
Supplement: Supplementary file 1 — Supplementary file1 (DOCX 18 KB) [file 592_2022_2005_MOESM1_ESM.docx]

**Search strategies**

**PubMed: 214 Results, 12.20.2021**

("MicroRNAs"[MeSH Terms] OR ("microrna s"[All Fields] OR "MicroRNAs"[MeSH Terms] OR "MicroRNAs"[All Fields] OR "microrna"[All Fields])) AND ("diabetes, gestational"[MeSH Terms] OR ("diabetes, gestational"[MeSH Terms] OR ("diabetes"[All Fields] AND "gestational"[All Fields]) OR "gestational diabetes"[All Fields] OR ("gestational"[All Fields] AND "diabetes"[All Fields] AND "mellitus"[All Fields]) OR "gestational diabetes mellitus"[All Fields]))

**Cochrane Library: 3 Results, 12.20.2021**

MeSH descriptor: [Diabetes, Gestational] explode all trees

MeSH descriptor: [MicroRNAs] explode all trees

**Embase: 379 Results, 12.20.2021**

'microrna'/exp AND 'pregnancy diabetes mellitus'/exp

**Supplementary Table 1. Quality assessment of included studies by Newcastle-Ottawa Scale**

| **NOS case-control**  **Study** | **Is the case definition adequate?** | **Representativeness of the cases** | **Selection of controls** | **Definition of controls** | **Comparability of cases and controls on the basis of the design or analysis** | **Ascertainment of intervention** | **Same method of ascertainment for cases and controls** | **Non-response rate** | **Total quality scores** |
| --- | --- | --- | --- | --- | --- | --- | --- | --- | --- |
| Pheiffer et.al.,2018 | ☆ | ☆ | ☆ | ☆ | ☆ | ☆ | ☆ | ☆ | 8 |
| Hocaoglu et.al.,2019 | ☆ | ☆ | ☆ | - | ☆ | ☆ | ☆ | ☆ | 7 |
| Gillet et.al.,2019 | ☆ | ☆ | ☆ | ☆ | - | ☆ | ☆ | ☆ | 7 |
| Hocaoglu et.al.,2020 | ☆ | ☆ | ☆ | ☆ | ☆ | ☆ | ☆ | ☆ | 8 |
| Deng et.al.,2020 | ☆ | ☆ | ☆ | ☆ | ☆ | ☆ | ☆ | ☆ | 8 |
| Wang et.al.,2019 | ☆ | ☆ | - | - | ☆ | ☆ | ☆ | ☆ | 6 |
